# Development and initial evaluation of a point-of-care educational app on medical topics in orthogeriatrics

**DOI:** 10.1007/s00402-015-2366-8

**Published:** 2015-12-08

**Authors:** Katrin Singler, Tobias Roth, Sacha Beck, Michael Cunningham, Markus Gosch

**Affiliations:** Department of Geriatrics, Klinikum Nürnberg, Paracelsus Private Medical University, Nürnberg, Germany; Institute for Biomedicine of Ageing, Friedrich-Alexander-Universität Erlangen-Nürnberg, Nürnberg, Germany; Department of Trauma Surgery, Medical University of Innsbruck, Innsbruck, Austria; Department of Geriatrics, Stadtspital Waid, Zurich, Switzerland; AO Education Institute, AO Foundation, Dübendorf, Switzerland

**Keywords:** Fragility fractures, Older adults, Orthogeriatrics, Education, Osteoporosis, Delirium, Anticoagulation, Perioperative pain

## Abstract

**Introduction:**

Research by AOTrauma’s orthogeriatrics education taskforce identified ongoing educational needs for surgeons and trainees worldwide regarding the medical management of older adults with a fracture. To address practicing surgeons’ preference for increased use of mobile learning, a point-of-care educational app was planned by a committee of experienced faculty. The goals were to deliver the app to surgeons, trainees, and other healthcare professionals, to measure usage, and to evaluate the impact on patient care.

**Materials and methods:**

The committee of geriatricians and surgeons designed and developed four modules on osteoporosis, delirium, anticoagulation, and pain based on published evidence and the content was programmed into mobile app formats. A registration form was integrated and a 14-question online evaluation survey was administered to users.

**Results:**

The AOTrauma Orthogeriatrics app was installed by 17,839 users worldwide between September 2014 and October 2015: Android smartphones (44 %), iPhones (32 %), iPads (15 %), Android tablets (9 %). 920 users registered and 100 completed the online evaluation: orthopedic/trauma surgeons (67 %), residents/fellows (20 %), and other professionals (13 %). Ratings for all aspects were 4 or higher on a 1–5 Likert scale (5 = Excellent). 80 % of evaluation respondents found the answer to their question or educational need on their last visit, and 26 of 55 respondents (47 %) reported making a change in an aspect of their management of patients as a result of their learning from the app.

**Conclusion:**

The orthogeriatrics app reached its intended audiences and was rated highly as a method of providing education to help improve patient care. Content input by experienced faculty and app improvements based on user feedback were key contributors to successful implementation.

## Introduction

A major increase in the availability and use of mobile applications (apps) for smartphones and tablet devices to deliver medical education has been reported over recent years, including tools for workplace-based and point-of-care learning [1–3]. Very few studies have been published to show the planned integration of these educational resources for surgeons and other physicians and the impact on learning, performance, and patient care [4–7]. Based on evaluation data and feedback from the implementation of a new curriculum over several years, the AOTrauma orthogeriatrics education taskforce decided their portfolio of face-to-face educational events, online/eLearning activities, and other resources could be enhanced [8–10]. The taskforce identified several competencies and performance gaps related to medical aspects of care in older adults with a fragility fracture that could be addressed in an educational app. They established a planning committee of geriatrician and surgeon faculty to develop a point-of-care learning app for surgeons and other healthcare professionals managing these patients.

Medical aspects of care, especially co-morbidities and complications make the treatment of fractures in older adults more challenging, and suboptimal management of these aspects is likely to cause increased rates of postoperative complications [11]. These issues are common to all systems of care, even within an interdisciplinary co-managed approach with a geriatrician and an orthopedic surgeon focused on in-hospital treatment. Education has also been identified as an area of need in a study of barriers to implementation of an organized geriatric fracture program [12]. In a 2012 global needs analysis, 20 % of practicing surgeons worldwide reported a need for more education in orthogeriatrics as well as a preference to have more mobile learning activities [13]. Reports in the literature at that time suggested the value of apps for orthopedic surgeons [14], and more recent studies indicate that the use of smart phone apps by both residents and consultants has now become widespread [15, 16].

This research study is an analysis of app installation statistics worldwide between September 2014 and October 2015, profiling information and needs assessment data from registered users, and evaluation ratings and feedback from surgeons and residents. The primary research questions are:

1. Did the app reach its target audiences and what were the reasons users installed it?
2. What questions and educational needs did users have when they accessed the app?
3. What aspects of patient care did users improve as a result of their learning from the app?

## Methods

### App design and development

**Fig. 1:**
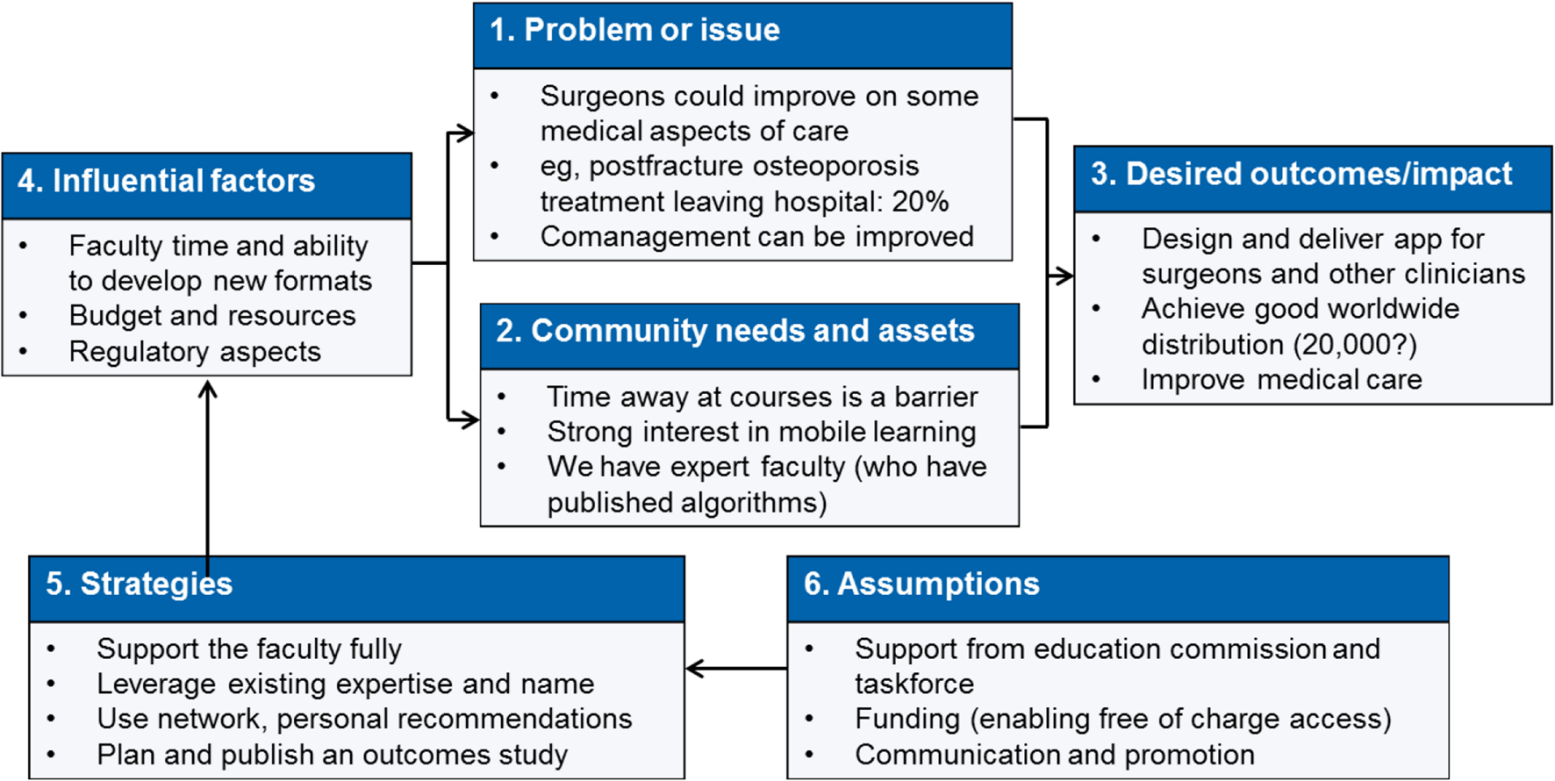
Program planning component of logic model for AOTrauma Orthogeriatrics App

**Fig. 2:**
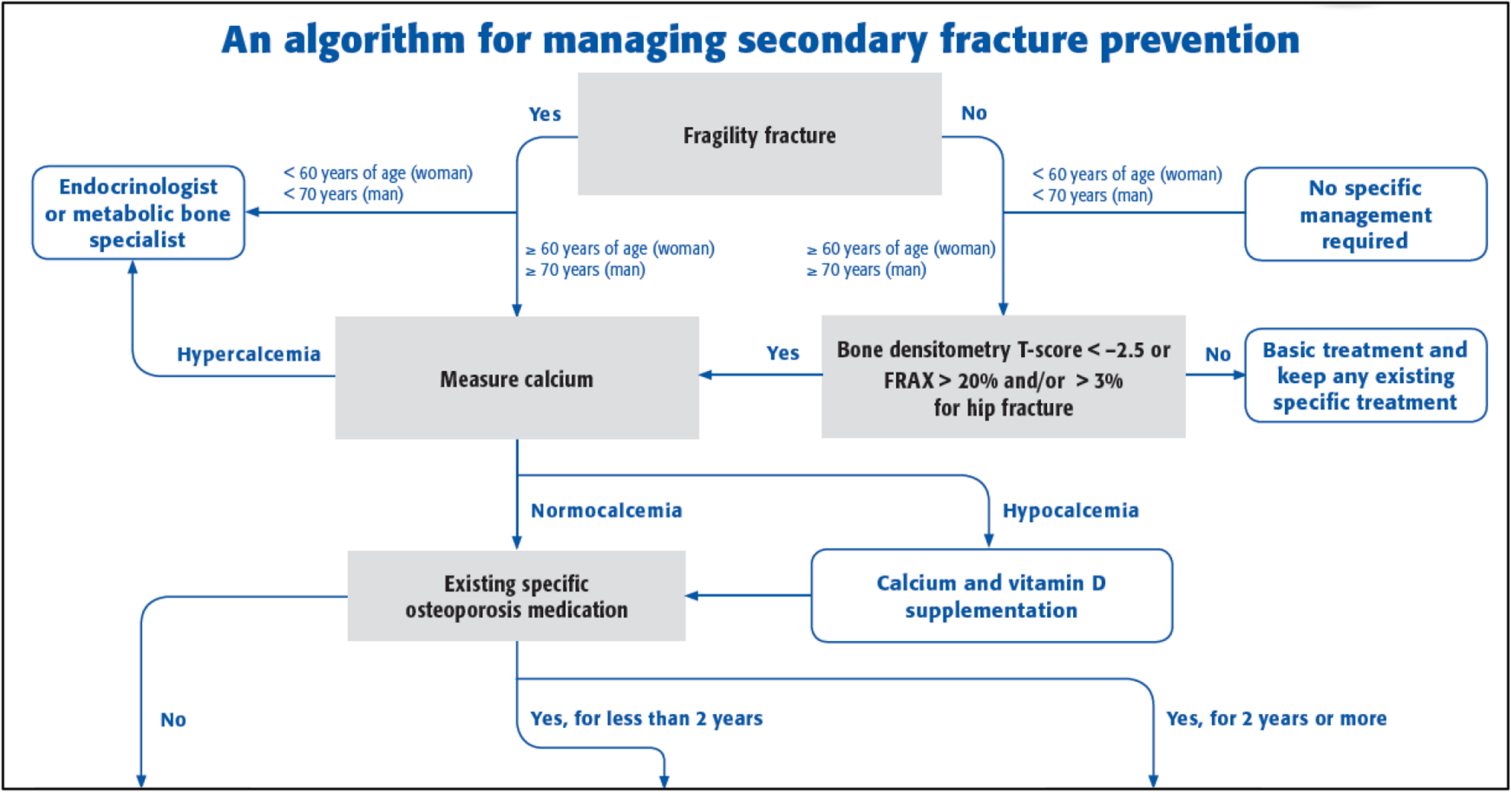
Part of overall pathway for osteoporosis module. ^†^Printable electronic versions of all pathways are available to users at: https://aotrauma.aofoundation.org/Structure/education/educational-programs/orthogeriatrics/Pages/mobile-apps.aspx

The planning committee for the orthogeriatrics app held two face-to-face meetings to analyze the available information and to identify learning outcomes from the curriculum that would be most appropriate. They decided to address gaps in knowledge, attitudes, and skills related to four medical topics: osteoporosis (secondary fracture prevention), anticoagulation, delirium, and pain; see Fig. 1. A review of needs assessment and commitment to change data from past courses confirmed these gaps and the committee decided to create a module for each topic based on an algorithm/pathway approach to the clinical situations and questions that surgeons and healthcare professionals are faced with in everyday practice, based on published pathways by Gosch et al. on secondary fracture prevention [17] and Wendl-Soeldner et al. on anticoagulation [18] and on further recent literature [19–22]. The structuring of content in these pathways would enable the user to quickly access information and evidence-based content in daily practice (an important aspect for point-of-care learning) and also to be able to work through the content in a more detailed manner for general education; see Fig. 2.

One main author was appointed to design the detailed pathway and content for each medical topic. The committee and a peer review process provided support and feedback. A pilot version was programmed and evaluated by 28 surgeons, residents, and geriatricians, and the authors analyzed the feedback [23]. Minor design and content changes were implemented and final quality assurance processes were completed. The app was launched in the iTunes and Google Play stores in September 2014 with the following description and disclaimer: The AOTrauma Orthogeriatrics App is an educational tool for healthcare professionals managing older adults with a fragility fracture. The primary audiences are surgeons and surgical trainees and the content is also appropriate for other physicians and healthcare professionals who are involved in the co-management of these patients. The app is for educational purposes only and the content is presented through clinical pathways for each medical topic. The app is NOT designed for making diagnostic or treatment decisions for any individual patient. The app was promoted at our educational courses, in newsletter items, and through review on external websites.

### Study design

Our research study was completed by analyzing app store data and by conducting two online questionnaires to gather: (1) Prospective profiling and intended use data from users who installed the app, and (2) Retrospective quantitative ratings and qualitative feedback from registrants who used the app.

## Materials

A seven-item registration form was designed to capture profiling information from users. A needs assessment using a gap analysis on four competencies was added. A 14-item evaluation questionnaire was designed to gather data on usage patterns and application in clinical practice.

## Methods

The registration process was integrated directly within the app as a voluntary option through a link to SurveyMonkey.com. The evaluation questionnaire was also programmed in SurveyMonkey. Registered users were invited by email to complete the evaluation process online, and a reminder was sent over a follow-up period of 3 weeks. The collection of evaluation data was continued until 100 responses were received. Installation data were extracted from the reporting tools within the iTunes and Google Play app stores.

### Data analysis

Data were compiled into graphs and tables showing descriptive statistics directly from SurveyMonkey and the analytics tools within the app stores, with some additional calculations after downloading to Microsoft Excel. Standard statistical analyses were conducted using these software tools. Open text responses were coded and themed using medical and educational categories.

This educational research project was approved by the Ethics Commission of the Faculty of Medicine at Friedrich-Alexander-University (FAU) Erlangen-Nürnberg, Germany. User registration within the app is a voluntary process and all invitations to complete the evaluation survey contained a message that participation was voluntary and for educational planning and research purposes, and that individual responses would remain anonymous and be grouped for data reporting and analysis.

## Results

**Fig. 3:**
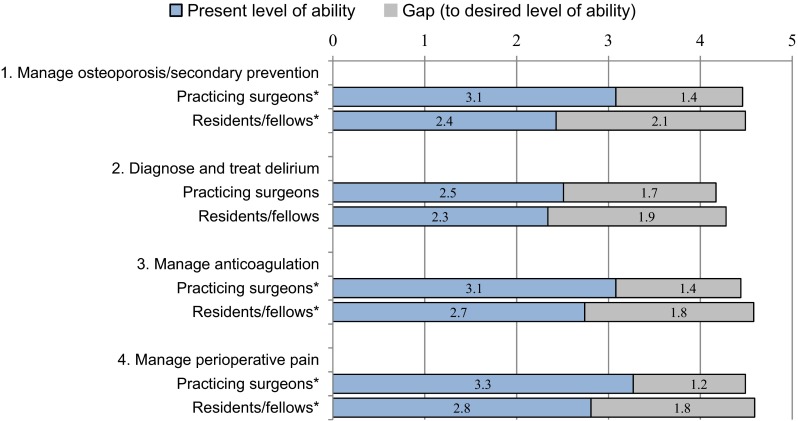
Self-reported gap scores (*n* = 162 surgeons and *n* = 68 residents/fellows) (1 = Low, 5 = High level of ability). *Significant difference in some categories of present level of ability between practicing surgeons and surgery residents/fellows (*p* = 0.05)

**Fig. 4:**
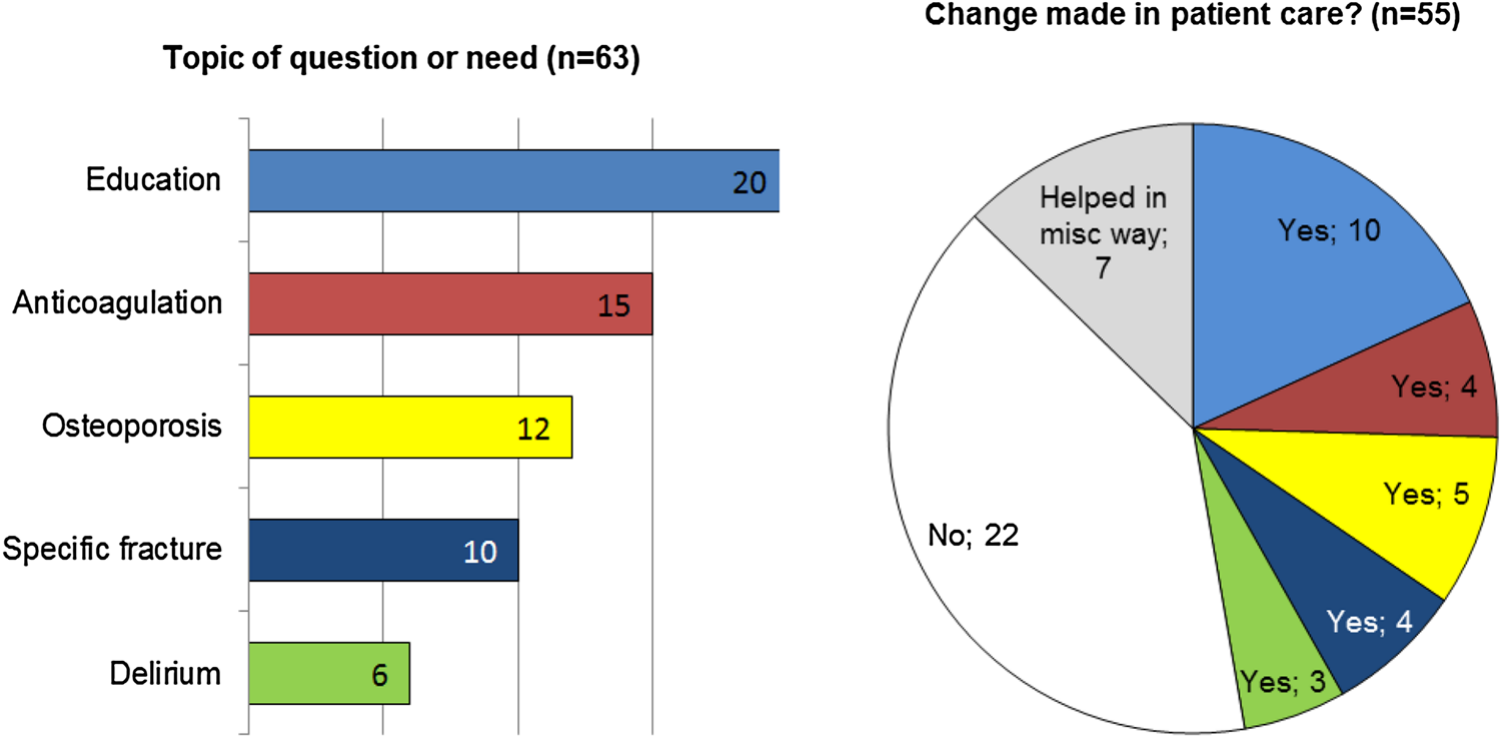
Categorized responses to “What was your specific question or educational need?” and “Did you make any change as a result of your learning from the app?”

During the 14 months following its launch, the AOTrauma Orthogeriatrics app was installed by 17,839 users from almost every country and on all four targeted smartphone and tablet devices; see Table 1. The lists of top 10 countries for numbers of downloads shows different patterns of use of the two main app stores (Google Play and iTunes), while the cumulative data show the most downloads were from India (8.5 %), USA (7.1 %), and Brazil (6.4 %). The top 10 countries accounted for almost half of the overall downloads, and the numbers of downloads from other countries varied greatly. 920 users completed the voluntary registration form within the app; see Table 2. Users reported the two main reasons they downloaded the app as “To aid decision making managing your patients” and “As an educational tool for yourself”, and more than one-third also reported they were “Interested in technology-based learning”. Self-reported gaps between present and desired level of abilities for the four medical competencies ranged between 1.2 and 2.1 for the 162 practicing surgeons and 68 residents/fellows who completed the needs assessment; see Fig. 3. Evaluation ratings for all aspects of the overall app and the individual modules were four or higher on a 1–5 Likert scale (5 = Excellent); *n* = 75, see Table 3. 80 % of respondents found the answer to their question or educational need on their last visit. 26 of 55 respondents (47 %) reported making a change in an aspect of their management of patients as a result of their learning from the app; see Fig. 4.

**Table 1:** Performance indicators for AOTrauma Orthogeriatrics App (Sept 2014–October 2015)

	iTunes app data	Google Play app data	Overall data
User installations (% of overall)	8300 (46 %)	9539 (54 %)	17,839
Smart phones (% of overall)	5700 (32 %)	7988 (44.8 %)	13,688 (76.7 %)
Tablet devices (% of overall)	2600 (14.5 %)	1551 (8.7 %)	4151 (23.3 %)
Retention rate (app not uninstalled)	Not available	60 %	63 % updated to V2
Current and highest ratings (users)	Not available	4.42, 4.53 (*n* = 180, 30)	–
Most downloads in a month	1380 (Sept 2014)	1104 (Sept 2014)	2484 (Sept 2014)
Fewest downloads in a month	306 (Aug 2015)	441 (Dec 2014)	816 (Aug 2015)
Downloads during most recent month	374 (Oct 2015)	706 (Oct 2015)	1080 (Oct 2015)
Top 10 countries (downloads)	USA (1011) China (731) Brazil (609) Germany (500) Mexico (462) India (292) UK (289) Spain (263) Italy (255) Netherlands (246)	India (1232) Egypt (624) Brazil (541) Mexico (526) Germany (417) USA (263) Argentina (243) Italy (231) Spain (228) Russia (228)	India (8.5 %) USA (7.1 %) Brazil (6.4 %) Mexico (5.5 %) Germany (5.1 %) China (4.2 %) Egypt (4.0 %) Spain (2.7 %) Italy (2.7 %) UK (2.5 %)

**Table 2:** Registered users and reasons for installing the AOTrauma Orthogeriatrics App (*n* = 872)

	Practicing surgeons	Surgery residents/fellows	Other healthcare professionals (various)
Number of registrants	586 (67 %)	176 (20 %)	110 (13 %)
Main reasons for downloading the app			
To aid decision making managing your patients	75 %*	67 %*	41 %
To aid decision making by other team members	26 %	26 %	14 %
As an educational tool for yourself	56 %*	75 %*	60 %
As an educational tool for others at hospital	25 %	22 %	18 %
Interested in technology-based learning	34 %	37 %	38 %

* Significant difference between practicing surgeons and residents/fellows (*p* = 0.05)

**Table 3:** Responses to evaluation questions regarding app ratings and usage patterns (*n* = 75)

App overall	1 (poor)	2	3 (average)	4	5 (excellent)	Mean	SD
Ease of installation	1	2	5	27	**40**	**4.37**	0.83
Information about authors	0	0	20	**31**	24	**4.05**	0.76
Interface, screen design	1	4	14	**29**	26	**4.01**	0.94
Navigation and ease of use	0	1	18	24	**29**	**4.13**	0.83
Overall content	0	2	19	24	**28**	**4.07**	0.87
Usefulness in clinical practice	0	1	18	**28**	26	**4.08**	0.81
Ease of access to information	0	1	11	25	**34**	**4.30**	0.78

Osteoporosis module	1 (poor)	2	3 (average)	4	5 (excellent)	Mean	SD
Content	0	2	12	23	**29**	**4.20**	0.84
Navigation pathway	0	3	13	21	**29**	**4.15**	0.89

Delirium module	1 (poor)	2	3 (average)	4	5 (excellent)	Mean	SD
Content	0	1	13	**17**	**17**	**4.04**	0.84
Navigation pathway	0	2	9	16	**20**	**4.15**	0.87

Anticoagulation module	1 (poor)	2	3 (average)	4	5 (excellent)	Mean	SD
Content	0	1	9	21	**28**	**4.29**	0.78
Navigation pathway	0	1	9	19	**29**	**4.31**	0.79

Times used during past week	0	1	2–5	6–10	10+	Mode	
Osteoporosis	16	**25**	**25**	4	3	**1, 2–5**	
Delirium	**30**	20	11	1	1	**0**	
Anticoagulation	17	20	**23**	3	4	**2–5**	

Average time per visit	1 min	2 min	3–5	6–10	10+ min	Mode	
Osteoporosis	10	11	**24**	11	7	**3–5**	
Delirium	8	10	**15**	5	2	**3–5**	
Anticoagulation	9	11	**19**	11	7	**3–5**	

The pain module was added after this evaluation

Bold text indicates the most frequent response(s)

## Discussion

The AOTrauma Orthogeriatrics App has been downloaded and used widely in all regions, especially in countries where it was heavily promoted by taskforce or commission members, in countries with a large population of AO surgeons, and in countries where apps are used more in general. Monthly downloads remain high due to continued promotion and the highest numbers of daily installations can be linked in many instances to promotion of the app through a newsletter item, email communication, or course.

The evaluation data and user ratings suggest that the design of the app modules around pathway algorithms based on clinical questions successfully reached and was well received by the intended audience of orthopedic trauma surgeon, trainees, and other healthcare professionals. The usage data supports the planned design of the app for: (1) point-of-care learning and (2) general education on the medical topics that were covered.

Several benefits of educational apps have been reported in previous studies: increased physician knowledge in managing patients with *S. aureus* bacteremia [4], increased confidence in managing patients with delirium [5], increased confidence in selecting depression treatment [6], and increased skills in chest tube insertion [7]. Our study adds to the growing body of literature in this new area of research and shows that some users were able to identify questions or gaps and then make improvements in some aspects of their care of patients as a result of their learning from the app. These self-reported improvements centered on improved assessment of patients, identification of treatment options, and recognition of appropriate indications. The gaps that were identified are important for the management of older adults, and the process of identifying the difference between the present level of ability and the desired level of ability for each competency is a widely used tool to gather this helpful information [8].

During development, challenges for the authors included the identification and integration of common questions and scenarios faced in daily practice, the provision of appropriate amounts of information on each “screen”, and the inclusion of adequate options to cover the possible pathways of care for each topic. Strategies to address the screen space issue and the intended dual use as a point-of-care learning tool and as a general educational tool included: moving background information to pop-up screens, providing shortcuts to tools and medication lists, presenting information as bullet lists, and the creation of an overview poster for each module to be used in conjunction with the app. Based on the ratings for the app overall and each of the modules, a high-quality experience seems to have been delivered to users.

The app has been downloaded by 10 or more users in more than 80 countries. Creating content for a global audience poses additional challenges (e.g., variability in the medications that are available, presence or absence of local or national guidelines, and differences in the roles of the members of the healthcare team in various systems of care). The authors and reviewers decided to focus on options that are common around the world and on key concepts and messages that could be considered and adapted by users in their local settings. A very important aspect of delivering the app was to clearly communicate that it is an educational tool only and that all users remain solely responsible for all clinical decisions they make.

The application of several concepts and frameworks from previous publications were very helpful for guiding the design and development phases: questions asked by physicians as the basis for needs assessment [24], an algorithm approach to designing a clinical decision-support tool [25], how physicians identify, assess, and utilize mobile medical applications in clinical practice [26], the Kellogg Logic Model for planning [27], and the development phases recommended by the US Agency for Healthcare Research and Quality (AHRQ) [28].

Six quality-assurance processes were integrated and are recommended for app projects: peer review, pilot testing, monitoring of feedback within the app, review of comments and ratings in the app stores and on websites, analysis of evaluation comments, technical testing of all operating systems and devices, and an annual review of content. The planning committee reviewed all peer and user comments and changes were made by the module authors during the development, pilot, and launch phases in response to the information gathered from each of these processes. This highlighted the importance of (1) having experienced faculty lead the design and content decisions, and (2) gathering user input and feedback to provide data to guide these decisions. The importance of advertising was highlighted by the increase in the numbers of daily installations following promotion at a course, in a newsletter item, or review on an external website. Based on continuous monitoring of feedback from users and peers, several potential enhancements to the app have been identified for future development, including the addition of new modules, links to article abstracts, integration of more assessment tools, and the creation of an online forum. The planning committee also agreed that an annual review of the modules is required in order to make sure the content is up to date. A full content review should also be conducted if any major new studies or guidelines are published and before all updates to the app (e.g., all content was reviewed during the month before version 2.0 was published in June 2015 to add the pain module).

Some of the main limitations of this study are: (1) the evaluation data represented 1 % of the total users who downloaded the app and may have been biased by a higher level of engagement from responses from our existing AOTrauma community, (2) the evaluation questions were not validated and represent a new approach in this relatively new field of research, and (3) the self-reported changes in practice were not verified and sometimes difficult to categorize. Future evaluation could include pre and post-app usage assessment questions, reevaluation of gap scores over time and with varying intensity of use, validation of the reported improvements in patient care through the review of patient charts, or the inclusion of a documented reflective process such as the one outlined in the Internet point-of-care learning section of the physician’s recognition award and credit system from the AMA.

## Conclusion

This research study supports the proposal that an educational app can be successfully integrated as a valuable component of a curriculum for surgeons and trainees for both point-of care and general learning. Reaching the intended target audience relies on appropriate communication and promotion, and the probability of retaining users is increased by delivering a high quality product with an easy-to-use design and interface. The faculty of content specialists and reviewers must lead the content decisions and be supported by a team for curriculum planning, instructional and interface design, and technical implementation.
